# Identification of dyslipidemia as a risk factor for sudden sensorineural hearing loss: A multicenter case‐control study

**DOI:** 10.1002/jcla.24067

**Published:** 2021-10-21

**Authors:** Xiaoqing Li, Binghua Chen, Xingxing Zhou, Fan Ye, Yumin Wang, Wangqiang Hu

**Affiliations:** ^1^ Department of Laboratory Medicine The First Affiliated Hospital of Wenzhou Medical University Wenzhou China; ^2^ Department of Laboratory Medicine Ningbo Medical Treatment Center Li Huili Hospital Ningbo University Ningbo China; ^3^ Department of Otolaryngology The First Affiliated Hospital of Wenzhou Medical University Wenzhou China

**Keywords:** case‐control study, dyslipidemia, sudden sensorineural hearing loss

## Abstract

**Background:**

Recently, several studies have reported an association between lipid profiles and sudden sensorineural hearing loss (SSNHL), yet there is considerable variability between the individual studies in defining the precise association between serum lipids levels and SSNHL. This study sought to identify a possible relationship between dyslipidemia and the prevalence and prognosis of SSNHL.

**Methods:**

A case‐control study was carried out at two independent medical centers, including 2,288 SSNHL patients and 2,288 healthy controls. Clinical characteristics and serum lipid parameters were assessed, including total cholesterol (CHOL), high‐density lipoprotein (HDL), low‐density lipoprotein (LDL), triglycerides (Trig), apolipoprotein AI (ApoAI), apolipoprotein B (ApoB), and lipoprotein a (Lpa). Multivariate logistic regression analysis was performed to assess the relationship between lipid profiles and SSNHL in the 4,576 subjects.

**Results:**

Significant differences were identified in several conventional serum lipid markers including CHOL, Trig, HDL, LDL, ApoAI, ApoB, and Lpa, between SSNHL patients and healthy controls. Serum ApoAI levels were significantly lower in patients with bilateral SSNHL compared to unilateral SSNHL. Binary logistic regression analysis revealed that higher levels of ApoB, LDL, Trig, and lower levels of ApoAI and HDL were all associated with an increased risk of SSNHL. After clinical characterization, multivariate analysis showed that only low levels of ApoB predicted likelihood of a recovery of more than 30 dB among patients with SSNHL.

**Conclusions:**

Serum lipids are associated with the incidence and prognosis of SSNHL. Identification of dyslipidemia may improve early evaluation and management of SSNHL risks.

## Graphical abstract

Binary logistic regression analysis of various lipids and SSNHL risk. Although CHOL and Lpa concentrations differed significantly between SSNHL patients and control subjects, neither the CHOL nor Lpa was related to SSNHL in multivariate analysis. Subjects with ApoB levels 5.54‐fold (95% CI 4.11–7.47) higher than that of healthy controls were more likely to have SSNHL pathology. Patients with abnormal LDL and Trig levels had a 10% (1.10 (1.03–1.18)) and 29% (1.29 (1.22–1.35)) increased odds of having SSNHL compared with healthy controls. Levels of ApoAI in patients with SSNHL were significantly lower (0.16 (0.13–0.21)) than that of control subjects. Conversely, HDL levels of control subjects were lower than those observed in patients with SSNHL (0.67 (0.56–0.80)).

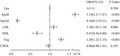

## 1 INTRODUCTION

Sudden sensorineural hearing loss (SSNHL) is a medical emergency, defined as sensorineural hearing loss of more than 30 dB, occurring within 3 days, on more than three consecutive frequencies of pure‐tone audiometry.^1^ The rapid hearing decline experienced with SSNHL is often idiopathic, and the prognosis for patients with SSNHL is hard to predict.^2^ Proposed SSNHL etiologies include vascular events, prothrombotic susceptibility, and inflammatory state.^3^, ^4^, ^5^, ^6^ Recently, several studies have reported an association between lipid profiles and SSNHL.^7^, ^8^, ^9^ However, there is considerable variability between the individual studies in defining the precise association between abnormal serum lipid levels and SSNHL occurrence.^3^, ^7^, ^8^, ^9^ However, conflicting reports do not support the conclusion that lipid profile is a risk factor for SSNHL development.^3^, ^10^, ^11^ Here, we aim to provide additional insights into the possible association between idiopathic SSNHL prevalence and serum lipids, using a case‐control controls study at two medical centers, as well as evaluate the role blood lipid levels play in the prognosis of patients with SSNHL.

## 2 MATERIALS AND METHODS

### 2.1 Participants

A total of 2,288 patients diagnosed with SSNHL and 2,288 control subjects were enrolled in the study at the First Affiliated Hospital of Wenzhou Medical University and Ningbo Medical Treatment Center Li Huili Hospital in China between 2015 and 2021. All patients diagnosed with SSNHL were hospitalized. The control subjects had normal bilateral hearing and no history of ear diseases. ENT examination and audiological evaluations were performed on both groups.

### 2.2 Blood lipid examinations

Blood lipid profile included total cholesterol (CHOL, mmol/L), high‐density lipoprotein (HDL, mmol/L), low‐density lipoprotein (LDL, mmol/L), triglycerides (Trig, mmol/L), apolipoprotein AI (ApoAI, g/L), apolipoprotein B (ApoB, g/L), and lipoprotein a (Lpa, mg/L). Blood lipid profiles were collected in all participants after hearing loss occurred and before treatment was initiated.

### 2.3 Exclusion criterion and clinical treatment

The exclusion criteria for idiopathic SSNHL patients included noise‐induced hearing loss, vestibular schwannoma, otitis media, neurologic disorders, Meniere's disease, patients using drugs with known ototoxic side effects, SSNHL or infections origin, or other major diseases (e.g., head trauma). All the patients were admitted to the department of otolaryngology and received clinical therapeutics according to the Guidelines for the Diagnosis and Treatment of Sudden Deafness (2015) recommendations.^12^

### 2.4 Hearing evaluation

The 2,288 SSNHL patients were evaluated by pure‐tone audiometry and had a measured hearing loss of 30 dB for at least three consecutive frequencies. Air and bone conduction at frequencies of 250 Hz, 500 Hz, 1 kHz, 2 kHz, 4 kHz, and 8 kHz were evaluated. Pure‐tone audiometry was evaluated before and post‐treatment. SSNHL outcome was calculated as the average of thresholds (dB HL) at three frequencies of 500, 1,000, and 2,000 Hz by additive pure‐tone audiometry. Treatment outcomes were evaluated in the last 200 SSNHL patients by audiometry results and recovery was defined as a more than 30 dB hearing improvement at the average of thresholds (dB HL) at three frequencies, with non‐recovery defined as less than 30 dB improvement.

### 2.5 Ethical considerations

The study was approved by The Ethics Committee at The First Affiliated Hospital of Wenzhou Medical University and Ningbo Medical Treatment Center Li Huili Hospital and performed in accordance with the Declaration of Helsinki. Oral informed consent was obtained from all participants.

### 2.6 Statistical analysis

Continuous data are shown as mean ± standard deviation, and categorical data are shown as frequencies or proportions. A prerequisite test for quantitative variables distribution was performed using Kolmogorov‐Smirnov test and Levene test. If independent variables had normal distribution and variance homogeneous, an unpaired t test was used, and if not, the non‐parametric Mann‐Whitney test was performed between two groups. For categorical data, a chi‐square test was performed. Binary logistic regression analysis was conducted to study the possible associations between lipid profiles and SSNHL, and an odds ratio (OR) with a 95% confidence interval (95% CI) was calculated. The statistical analysis was performed using SPSS version 18.0 for Windows (SPSS Inc.). A two‐sided *p* value < 0.05 was considered statistically significant.

## 3 RESULTS

### 3.1 Baseline patient clinical characteristics and serum lipid profiles

Patient characteristics based on SSNHL category are shown in Table 1. For all patients in the study, the proportion of males was 52.3% in the SSNHL subjects and 52.4% in the control subjects (*p *= 0.929). The average age at diagnosis of patients was 49.7 years for SSNHL patients and 51.0 years for controls (*p *= 0.092), with a range of 9–87 years. Of the SSNHL patients, 2,212 (96.7%) suffered from unilateral SSNHL and 76 (3.3%) were diagnosed with bilateral SSNHL. In the cases of unilateral SSNHL, the hearing loss was on the left side in 51.4% of patients compared to 45.3% of the cases on the right side.

**TABLE 1:** Patient clinical characteristics and serum level of lipid profile

Variables	SSNHL subjects (*n* = 2,288) mean ± SD	Control subjects (*n* = 2,288) mean ± SD	*p* value
Age (year)	49.7 ± 15.3	51.0 ± 13.4	0.092
Gender, *n* (%)			0.929
Male	1,197(52.3)	1,200(52.4)	
Female	1,091(47.6)	1,088(47.6)	
Blood lipids			
CHOL, mmol/L	5.24 ± 1.16	5.29 ± 1.04	0.046
Trig, mmol/L	1.99 ± 1.71	1.55 ± 1.26	<0.001
HDL, mmol/L	1.35 ± 0.34	1.40 ± 0.35	<0.001
LDL, mmol/L	2.96 ± 0.93	2.90 ± 0.81	0.021
ApoAI, g/L	1.23 ± 0.28	1.36 ± 0.30	<0.001
ApoB, g/L	0.88 ± 0.23	0.82 ± 0.20	<0.001
Lpa, mg/L	202.90 ± 234.70	214.50 ± 248.40	0.002

Abbreviations: ApoAI, apolipoprotein AI; ApoB, apolipoprotein B; CHOL, total cholesterol; HDL, high‐density lipoprotein; LDL, low‐density lipoprotein; Lpa, lipoprotein a; SD, standard deviation; Trig, triglycerides.

### 3.2 Serum lipid profiles between bilateral and unilateral SSNHL patients versus healthy controls

**FIGURE 1:**
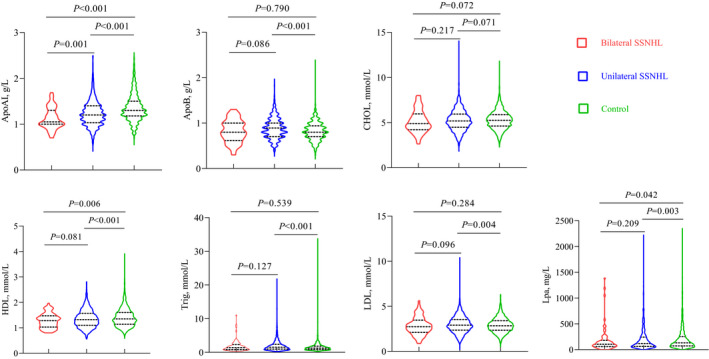
Selected lipid levels in SSNHL patients and controls

As shown in Table 1, CHOL, HDL, ApoAI, and Lpa concentrations were higher in the control subjects than in the SSNHL subjects. The mean CHOL concentration of the control subjects was slightly increased compared to the SSNHL subjects (5.29 ± 1.04 mmol/L vs. 5.24 ± 1.16 mmol/L; *p *= 0.046). The concentration of ApoAI in the bilateral SSNHL subjects was significantly lower than the unilateral SSNHL group (1.12 ± 0.23 g/L vs. 1.23 ± 0.28 g/L; *p *= 0.001) (Figure 1), yet no significant differences between bilateral SSNHL and unilateral SSNHL group were found for other lipid parameters including CHOL, HDL, and Lpa concentrations. Aside from CHOL, ApoAI, HDL, and Lpa values were significantly higher in the control subjects compared to the unilateral SSNHL patients.

Concurrently, plasma levels of Trig, LDL, and ApoB were significantly higher in patients with SSNHL compared to healthy controls (Table 1). Yet, no significant differences between bilateral SSNHL and unilateral SSNHL groups in Trig, LDL, and ApoB levels were observed (Figure 1). Trig, LDL, and ApoB values also did not differ significantly between bilateral SSNHL and control subjects.

### 3.3 Association between SSNHL incidence and lipid profiles

**FIGURE 2:**
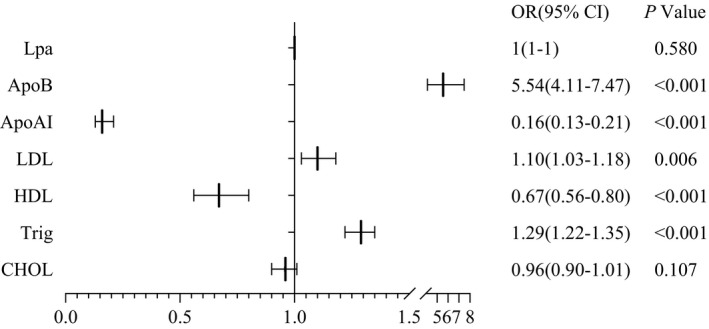
Binary logistic regression analysis of various lipids and SSNHL risk

The association between SSNHL prevalence and lipid profiles is shown in Figure 2. Although CHOL and Lpa concentrations differed significantly between SSNHL patients and control subjects, neither the CHOL nor Lpa was related to SSNHL in multivariate analysis (Figure 2). Subjects with ApoB levels 5.54‐fold (95% CI 4.11–7.47) higher than that of healthy controls were more likely to have SSNHL pathology. Patients with abnormal LDL and Trig levels had a 10% (1.10 (1.03–1.18)) and 29% (1.29 (1.22–1.35)) increased odds of having SSNHL compared with healthy controls. Levels of ApoAI in patients with SSNHL were significantly lower (0.16 (0.13–0.21)) than that of control subjects. Conversely, HDL levels of control subjects were lower than those observed in patients with SSNHL (0.67 (0.56–0.80)).

### 3.4 Relationship between lipid profiles and SSNHL prognosis

**FIGURE 3:**
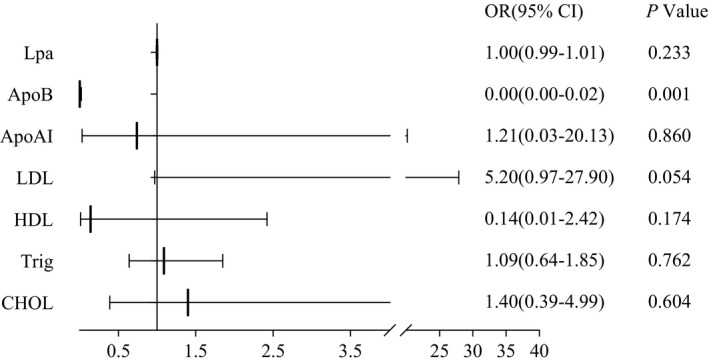
Multivariable logistic regression analysis of SSNHL patient lipids and recovery of more than 30 dB

Multivariate logistic regression analysis revealed that ApoB level (0.00 (0.00–0.02)) was an independent risk factor for poor prognostic outcomes during the follow‐up (Figure 3). SSNHL patients with a lower ApoB were more likely to recover by more than 30 dB (*p *= 0.001) (Figure 3). We found no significant difference in Lpa, ApoAI, LDL, HDL, Trig, and CHOL levels between patients recovering more than 30 dB and those recovering less than 30 dB (*p *> 0.05).

## 4 DISCUSSION

Sudden sensorineural hearing loss, a relatively common disease, is typically idiopathic.^13^ Several studies have proposed a possible association between lipid profiles and SSNHL.^7^, ^8^, ^9^, ^14^ Yet, there is considerable variability among these studies, and the exact relationship between serum level of lipid profile and SSNHL remains elusive. Limitations of these studies included poor control subject selection, as well as single centers and low patient numbers.^4^, ^7^, ^8^

To address this, we performed a large age‐ and sex‐matched control study in two medical centers that included 2,288 SSNHL patients and 2,288 healthy controls. We focused on CHOL, Trig, HDL, LDL, ApoAI, ApoB, and Lpa serum levels, which have been suggested to be a risk factor for SSNHL.^15^, ^16^, ^17^

We found SSNHL patients exhibited higher concentrations of the Trig, LDL, and ApoB compared to healthy controls. Conversely, CHOL, HDL, ApoAI, and Lpa were significantly lower in SSNHL patients compared to healthy controls. Binary logistic regression analysis identified HDL, Trig, LDL, ApoB, and ApoAI levels as independent risk factors for SSNHL.

Many studies have reported that dyslipidemia may contribute to the clinical events of SSNHL.^9^, ^14^, ^18^, ^19^, ^20^ Quaranta et al.^19^ showed that CHOL concentrations can be a prognostic factor for recovery in SSNHL and should be assessed routinely in this patient population. Similarly, in a case‐control study, Lee et al.^9^ reported that elevated CHOL and Trig levels are significantly associated with the development of SSNHL, but there is no difference in LDL levels between the SSNHL and control groups. On the contrary, Kaneva et al.^8^ reported that dyslipidemia of conventional parameters, including CHOL, Trig, and HDL could not be used to differentiate SSNHL and healthy controls. We found that HDL, Trig, and LDL are independent risk factors for SSNHL, whereas CHOL and Lpa levels are not associated with SSNHL.

The cochlea is very sensitive to alterations in blood circulation, resulting from its terminal artery blood supply without collateral circulation.^21^ Thus, a prominent theory purports abnormal cochlea blood supply may contribute to hearing impairment, and cochlear ischemia has been proposed as the most prominent cause of SSNHL.^22^ Blood viscosity elevations due to dyslipidemia can disturb the cochlear microcirculation. Thus, higher concentrations of blood lipids have been identified as a primary risk factor of SSNHL and atherosclerosis (AS). Inner ear blood supplies could be decreased by AS of the cochlear vessels, and thus, inner ear damage may occur. Vascular damage can lead to cochlear ischemia and hypoxia and facilitate hearing impairment.^9^ Numerous studies demonstrate that lipoprotein lipid oxidation product (LOP) transport is associated with risk of developing AS. High levels of LOP carried by LDL are positively correlated with AS, and high LOP levels in HDL appear to negatively correlated with AS.^23^ It is well accepted that high serum concentrations of LDL play an important role in the onset and progression of AS. In contrast, HDL has anti‐inflammatory and anti‐oxidative effects, which is important for preventing AS.^24^, ^25^ High concentrations of Trig could also cause disturbances in cochlear microcirculation^26^ and may play a key role in the pathogenesis of SSNHL.^27^

Our data also show that higher serum ApoB levels and lower serum ApoAI levels are tightly associated with increased SSNHL incidence. Weng et al.^15^ also demonstrated that LDL and ApoB concentrations might be important factors in the pathogenesis of SSNHL. Cholesterol balance, defined as the ApoB/ApoAI ratio, has repeatedly been shown to be a better marker than lipoproteins.^28^, ^29^, ^30^ A high ApoB/AI ratio is related to intracranial atherosclerotic stenosis,^31^ a potential mechanism by which the cochlea is vulnerable to microvascular ischemia.

Apolipoprotein AI was found to be significantly higher in patients with unilateral SSNHL than in those with bilateral SSNHL. Since high serum ApoAI levels affect blood supply, they may damage cochlear neural cells.^32^ Similarly, a rise in the ApoB/ApoA‐I ratio in patients with SSNHL suggests an increasingly atherogenic lipid profile.^8^ Plasma ApoB and ApoAI are also reported to be stronger risk factors for coronary artery disease (CAD) than LDL and HDL.^33^ Zhang and colleagues showed that the average age of onset for unilateral SSNHL subjects was significantly younger than the bilateral SSNHL subjects.^4^ We suspect that this is because older SSNHL patients were more vulnerable to CAD.^34^

After clinical treatment, ApoB levels of SSNHL patients were significantly lower in those that recovered more than 30 dB compared to those that recovered less than 30 dB. Multivariate analysis also showed that only ApoB was an independent prognostic factor for SSNHL, suggesting that lower ApoB may be a good prognostic marker in SSNHL patients. Correction of dyslipidemia in SSNHL patients is thought to improve hearing, as prior studies have suggested that lipid profiles are correlated with the initiation of impaired hearing and its prognosis.^35^

## 5 CONCLUSIONS

We found that patients with SSNHL had higher serum LDL, ApoB, and Trig. Lower HDL and ApoAI levels were correlated with increased incidence of SSNHL. ApoB appears to be a positive prognostic factor in SSNHL.

## CONFLICT OF INTEREST

The authors indicate no potential conflicts of interest.

## AUTHOR CONTRIBUTIONS

Xiaoqing Li designed the research study and wrote the paper; Binghua Chen performed the acquisition of data and analyzed and interpreted the data; Xingxing Zhou analyzed and interpreted the data; Fan Ye administered the technical, clinical evaluation, and documentation; Yumin Wang reviewed and revised the manuscript; Wangqiang Hu wrote, reviewed, and revised the manuscript; all authors read and approved the final manuscript.

## Data Availability

The data that support the findings of this study are available from the corresponding author upon reasonable request.
